# Chloroquine/hydroxycloroquine and COVID-19: need to know more about

**DOI:** 10.2217/fmb-2020-0247

**Published:** 2020-11-16

**Authors:** Antonio Cassone, Licia Iacoviello, Roberto Cauda

**Affiliations:** ^1^Polo d'Innovazione della Genomica, Genetica e Biologia, University of Siena; ^2^Department of Epidemiology & Prevention, IRCCS Neuromed, Pozzilli (IS), Italy; ^3^Department of Medicine & Surgery, University of Insubria, Varese 21100, Italy; ^4^Department of Healthcare Surveillance & Bioethics, Catholic University Sacred Heart, Division of Infectious Diseases, Fondazione Policlinico A Gemelli IRCCS, Roma 00100, Italy

**Keywords:** anti-inflammatory, antiviral, COVID-19, HCQ dosage, hydroxychloroquine, immunomodulation, observational studies, randomized controlled trials, SARS-CoV-2

Chloroquine and its more soluble hydroxylated derivative (CQ/HCQ), are members of the quinoline class of compounds which have long been used in the treatment of malaria and inflammatory disorders such as rheumatoid arthritis and lupus erythematosus (recently reviewed in [1]). They are generally safe, easy to administer and cheap compounds. Since an initial report from Gao *et al.* [2], these two old drugs have also become the subject of an intense research activity and clinical use for their presumed prophylactic and/or therapeutic efficacy in patients with COVID-19, the pandemic disease caused by the newly emerged coronavirus SARS-CoV-2 (www.who.org). The basic rationale for repurposing CQ/HCQ for COVID-19 treatment stems from an initial hypothesis, raised by some of us and derived from studies on anti-HIV therapy [3], that CQ could inhibit SARS-CoV-2 [4,5], that was later verified [6] and has now been confirmed with the new coronavirus [7,8]. For this and the lack of specific anti-COVID-19 therapies and vaccines, CQ/HCQ were soon proposed and included in Chinese guidelines for COVID-19 treatment, though HCQ is no longer included [9].

In some early, anecdotal reports, CQ/HCQ were considered to be effective, particularly in early stages of the disease or as prophylaxis (systematically reviewed in [10]). To achieve a better therapeutic success some authors have proposed associating these drugs with azythromicin, an antibacterial macrolide with some antiviral properties [11,12]. Most of the published results on anti-COVID-19 activity of CQ/HCQ are from small, poorly controlled and nonrandomized clinical trials, with erratic and contrasting results, as discussed in [13]. Despite the absence of positive indications from large, well-conducted studies and randomized clinical trials (RCT), these drugs became very popular and widely used by clinicians. Quite inappropriately, they have also been politically-charged, being sponsored as game-changers by important political men and heads of government, and this greatly contributed to unwise use and shortage of CQ/HCQ [14].

An apparent turning point was the publication of the results from a large, observational and multinational RCT which reported lack of efficacy of HCQ against COVID-19 patients, both as therapeutic and as prophylactic agent [15–17]. Instead, the potential of the drug to cause cardiac toxicity was highlighted in some studies, also leading to the premature suspension of the HCQ arm of the trials [16,17]. One of these studies was subsequently retracted [18], and another one dealing with HCQ prophylaxis had notable limitations, including the lack of consistent diagnosis of SARS-CoV-2 in treated patients [19]. Despite these uncertainties, the results of these studies ingenerated, as a whole, the conclusion that CQ/HCQ were inefficacious as anti-SARS-CoV-2 agents and could be harmful in some patients. It is now a common experience of those performing COVID-19 clinical studies that the editors of major medical journals have become very reluctant to publish something dealing with CQ/HCQ and COVID-19 unless the conclusion confirms the RCT data, as seen in several recent meta-analyses [20–22].

Nonetheless, interest in these two drugs has remained alive as witnessed by the numerous (more than 100 worldwide), still ongoing clinical trials [23]. While the results of these trials are eagerly expected, two bodily, retrospective observational studies (ROS) have appeared, authored by Italian and Belgian investigators [13,24]. Together these two multicenter, nationwide studies have enrolled more than 10,000 hospitalized subjects with confirmed diagnosis of SARS-CoV-2 infection and treated with or without HCQ and standard supportive therapy as control. Cumulatively, around 7000 patients received HCQ at low (2400 mg) or moderate (maximum 4500 mg) dosage in almost 150 hospitals representing the whole hospital distribution over the national territory in Belgium and 33 centers in Italy, in other words, the major, biggest hospitals dealing with COVID-19 patients in the various Italian regions. Importantly, both studies reported a similar, substantial decrease of in-hospital mortality and no safety concerns in the HCQ-treated with respect to the mortality of HCQ-untreated, COVID-19 patients, after 13 days of follow-up (the Italian study) or until hospital discharge (24 days, the Belgian study). In a subgroup analysis of the Italian study which assessed HCQ effectiveness according to the level of a C-reactive protein on patient admission, a higher decrease of mortality in HCQ recipients with high level of this acute-phase inflammatory protein was detected [13].

The results of both studies do not allow a firm conclusion on the therapeutic efficacy of HCQ in COVID-19 patients because of the known ROS limitations, mainly their retrospective nature and the consequent lack of patient randomization. This bias cannot be totally eliminated by even extensive statistical corrections. This limitation being accounted for, the high number of treated patients and controls, coupled with careful data and subgroup analysis make the findings of these two studies quite valuable. They appear to be representative of a real world HCQ treatment of unselected, hospitalized patients as well as a faithful description of COVID-19 patients treated with HCQ in two European countries with high standards of public health and medical care. Although the data cannot be taken as indicators of a solid change in the future treatment of COVID-19, they should be considered for planning novel, well prepared and conducted RCT.

In this scenario, and as relevant tool for planning future trials, we point out here the low – moderate total HCQ dosage used in the Belgian and Italian studies, respectively, and the limited time (5–7 days) of treatment. These dosages are much lower than those used in the aforementioned RCT, particularly a third of that used in the RECOVERY trial [17]. The choice of a loading dose of 800 mg the first day of treatment and a maintenance dose of 400 mg was based on previous reports about how balancing treatment efficacy and good safety profile, as from pharmacokinetics modeling [25,26]. This is also the loading dose recommended for antimalarial treatment [25,26]. In the Belgian study, the HCQ dosage was particularly low (2400 mg total), nonetheless, the COVID-19 mortality was reduced regardless of the timing from symptoms onset to diagnosis and initiation of HCQ treatment [24].

Literature examination of a critical aspect such as the HCQ dosing in COVID-19 patients does not seem particularly helpful as in many studies there appears to be a varied selection of drug dosage regimen without any attempt to justify or optimize it. Although the difficulties of estimating HCQ tissue levels from plasma concentrations are well known and have recently been highlighted [26,27], it remains unclear why in the RCT studies such a high dosage of HCQ was used. We assume it was selected in order to maximize the direct antiviral activity of the drug as this activity was realistically taken as rationale of HCQ trial (see above). Possibly, those high doses reached values that have favored emergence of side effects without achieving antiviral benefits. This matter is also greatly complicated by the number of different mechanisms that have been postulated to mediate CQ/HCQ activity, going from inhibition of one or more steps of SARS-CoV-2 replication to immunomodulatory and anti-inflammatory effects, likely each mechanism with a different dose–activity relationship [25]. Both ROS discussed above attribute the limited but substantial benefit of HCQ treatment of COVID-19 patients to the immunomodulatory rather than to the direct antiviral activity of HCQ that is unlikely achieved by the drug dosage used in those studies, unless to admit a synergistic effect between antiviral and immunomodulatory HCQ activities [25,26]. Notably, anti-SARS-CoV-2 activity of HCQ, which has been largely confirmed *in vitro* with Vero cells [7,8,28], was not found using pneumocytes, alveolar lung cell lines and some animal models [28,29]. If immunomodulation/anti-inflammatory activity is the dominant or major mechanism of HCQ protection in COVID-19 patients, the high dosages used in the RCT studies could be unfit to stimulate a protective HCQ effect. In fact, nonsigmoidal, bell-shaped dose-response curves are not rare to obtain with drugs having complex immunomodulatory effects, with multiple-binding sites or cellular and organ targets, that just appears to be the case in point with CQ/HCQ.

It remains highly speculative to assume that one of, or the reason for, the anti-COVID-19 activity of HCQ is the use of a low-moderate drug dosage because of the lack of large studies directly comparing low-moderate with high HCQ treatment doses. We have found only one small randomized, double-blind study where severely ill COVID-19 patients were treated with high (12 g) or low (2.7 g) chloroquine doses for 10 days, and their mortality compared [30]. In that study, the number of patients were low (81) and the difference in lethality was not considered to be statistically significant, nonetheless, 16 out of 41 and 6 out of 40 patients died in the high and low dosage group, respectively.

Overall, the potential therapeutic or prophylactic activity of CQ/HCQ remains undetermined. The results of the numerous ongoing trials will possibly be of help. At any rate, it is our opinion that what we know so far, if correctly examined in all aspects and characteristics relevant to patients, HCQ usage and COVID-19 illness, would justify novel, correctly-designed and executed RCT, paying particular attention to the HCQ dosage and treatment duration. The abandonment of such a safe and cheap drug as HCQ would seem to be unjustified before new, knowledge-updated and oriented trials and until a specific, potent anti-SARS-CoV-2 therapeutic or vaccine becomes available. In essence, we still need to know more about CQ/HCQ and COVID-19.
